# Study on the Anti-Inflammatory Mechanism of Coumarins in *Peucedanum decursivum* Based on Spatial Metabolomics Combined with Network Pharmacology

**DOI:** 10.3390/molecules29143346

**Published:** 2024-07-17

**Authors:** Zeyu Li, Qian Li

**Affiliations:** State Key Laboratory of Aridland Crop Science, College of Agronomy, Gansu Agricultural University, Lanzhou 730070, China; lizy092023@163.com

**Keywords:** *Peucedanum decursivum* (Miq.) Maxim, coumarin, mass spectrometry imaging, network pharmacology, molecular docking

## Abstract

*Peucedanum decursivum* (Miq.) Maxim (*P. decursivum*) is a traditional Chinese medicinal plant with pharmacological effects such as anti-inflammatory and anti-tumor effects, the root of which is widely used as medicine. Determining the spatial distribution and pharmacological mechanisms of metabolites is necessary when studying the effective substances of medicinal plants. As a means of obtaining spatial distribution information of metabolites, mass spectrometry imaging has high sensitivity and allows for molecule visualization. In this study, matrix-assisted laser desorption mass spectrometry (MALDI-TOF-MSI) and network pharmacology were used for the first time to visually study the spatial distribution and anti-inflammatory mechanism of coumarins, which are metabolites of *P. decursivum*, to determine their tissue localization and mechanism of action. A total of 27 coumarins were identified by MALDI-TOF-MSI, which mainly concentrated in the cortex, periderm, and phloem of the root of *P. decursivum*. Network pharmacology studies have identified key targets for the anti-inflammatory effect of *P. decursivum*, such as TNF, PTGS2, and PRAKA. GO enrichment and KEGG pathway analyses indicated that coumarins in *P. decursivum* mainly participated in biological processes such as inflammatory response, positive regulation of protein kinase B signaling, chemical carcinogenesis receptor activation, pathways in cancer, and other biological pathways. The molecular docking results indicated that there was good binding between components and targets. This study provides a basis for understanding the spatial distribution and anti-inflammatory mechanism of coumarins in *P. decursivum*.

## 1. Introduction

*Peucedanum decursivum* (Miq.) Maxim (*P. decursivum*) is a widely used medicinal plant in the Apiaceae family, and its dry root is used as a medicine. It was first recorded in a classic called *Ming Yi Bie Lu* by Tao Hongjing [1]. *P. decursivum* prefers mild climates and mostly grows on sunny hillsides. It has a very wide distribution throughout the world, being present not only in China but also in Japan, Korea, India, etc. [2]. It has the effects of dispelling wind, clearing heat, and resolving phlegm [3]. *P. decursivum* mainly contains coumarins and volatile oils [4]. Coumarins in *P. decursivum* include nodakenin, decursin, imperatorin, and others. Modern pharmacological studies have shown that coumarins have anti-inflammatory [5], antiviral [6], antibacterial [7], anticoagulant [8], anticancer [9], anti-asthma [10], anti-osteoporosis [11], and neuroprotective effects [12].

Mass spectrometry imaging (MSI) can map the spatial distribution characteristics of the components of a sample. It combines mass spectrometry ion-scanning technology with professional image processing software to directly analyze biological tissue sections and generate two-dimensional ion density maps of compounds with arbitrary mass-to-charge ratios (*m*/*z*), thereby enabling a rapid and comprehensive analysis and research on the composition, relative abundance, and spatial distribution of substances in cells or tissues [13]. MSI has the advantages of high sensitivity, high spatial resolution, high degree of visualization, and low risk of contamination and degradation [14]. MALDI-TOF-MSI is the most widely used MSI method [15] that can visualize and analyze the chemical composition and spatial distribution characteristics of samples [16,17,18]. Kuo and his colleagues [19] first proposed a method based on MALDI-MSI and molecular network analysis to study plant metabolites. They used agarwood stems as slice materials and LC-MS and MALDI-MSI to analyze and track agarwood metabolites and found that agarwood secondary metabolites were concentrated in the resin. MSI can be used to study the spatial distribution characteristics of secondary metabolites in medicinal plants [20], their synthesis and transport pathways [21,22], accumulation patterns [23,24], and involvement in plant stress defense [25]. It provides new ideas for the specific research on medicinal plant components and the development of quality and safety testing.

Network pharmacology is based on the theory of systems biology. It uses the construction of biological networks as a method, high-throughput omics data, various databases, and the literature as a basis, and computer science and technology as the main scientific and technological means to conduct comprehensive and holistic analyses of the efficacy, toxicity, bioavailability, and mechanism of action of new drugs [26]. Network pharmacology reveals and analyzes the multi-level and multi-angle biological network relationships between “drugs, genes, targets, and diseases” to predict the possible mechanism of action of drugs and provide important reference for discovering their pharmacological efficacy [27]. Jiang [28] combined MSI with network toxicology to explore the potential targets and metabolic mechanisms of hepatotoxicity of *Polygonum multiflorum* Thunb component D, and the binding activity of toxic components and core targets were matched by molecular docking.

Therefore, the powerful combination of MSI and network pharmacology provides detailed information about the metabolites of medicinal plants and can constitute a platform for the determination of the pharmacological mechanisms of the bioactive metabolites of medicinal plants. In this research, MSI was used to determine the distribution of coumarins in the roots of *P. decursivum*, and network pharmacology was used to explore the anti-inflammatory mechanism of *P. decursivum*. A comprehensive analysis of *P. decursivum* was conducted to provide a reference for the quality evaluation and pharmacological application of *P. decursivum*.

## 2. Results and Discussion

### 2.1. Selection of the Optimal Thickness of Frozen Sections

**Figure 1:**
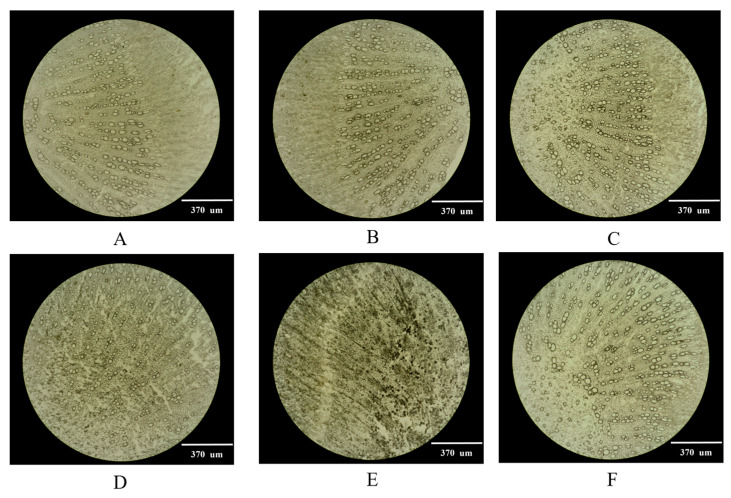
Microscopic observation of frozen sections of different thicknesses of *P. decursivum*. (**A**) 20 µm (100×), (**B**) 25 µm (100×), (**C**) 30 µm (100×), (**D**) 35 µm (100×), (**E**) 40 µm (100×), (**F**) 30 µm (100×).

The thickness of a tissue slice could affect its integrity. If the slice is broken, it will be impossible to obtain a complete image [29]. Complete and clear tissue sections are the key to obtaining high-quality images [30]. The root of *P. decursivum* was cut into slices with thicknesses of 20 µm, 25 µm, 30 µm, 35 µm, and 40 µm, whose integrity and expansion were observed in bright field using an upright and inverted fluorescence microscope. The results showed that when the slice thickness was less than 30 µm, the tissue was severely fragmented, and slicing was more difficult. When the slice thickness exceeded 30 µm, the slice was too thick, and the field of view was dark, reducing the clarity of the observation, although the slice integrity and stretchability were high. Therefore, 30 µm was selected as the slice thickness for the root of *P. decursivum* (Figure 1).

### 2.2. Fluorescence Imaging of the Distribution of Coumarins in the Root of P. decursivum

**Figure 2:**
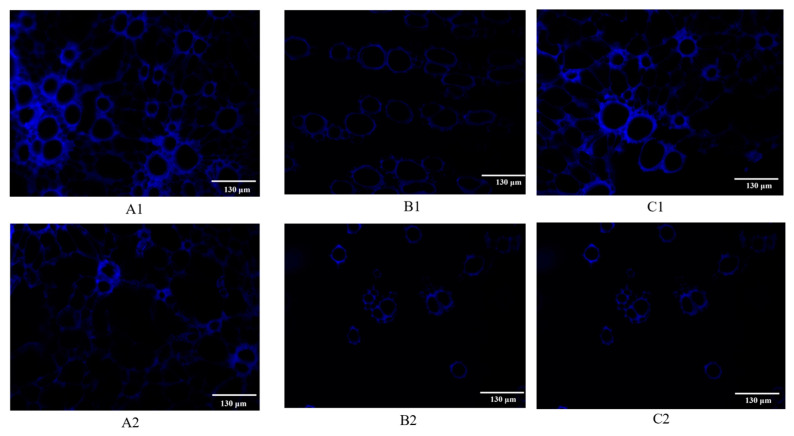
Microstructure of and coumarin localization in the root of *P. decursivum*. (**A1**) Cork layer, cork inner layer, phloem; 200×, (**A2**) ethanol-treated cork layer, cork inner layer, and phloem; 200×, (**B1**) phloem; 200×, (**B2**) ethanol-treated phloem; 200×, (**C1**) xylem, 200×, (**C2**) ethanol-treated xylem, 200×.

The frozen sections of *P. decursivum* were observed using the DAPI channel of an upright and inverted fluorescence microscope. The results showed that the cork layer, the inner cork secretory cavity, the phloem secretory duct, and the xylem emitted blue fluorescence, but it was difficult to determine the location of coumarins in the sections of *P. decursivum*. Therefore, taking advantage of the fact that coumarins are easily soluble in organic solvents, an ethanol treatment was applied. After the slices of *P. decursivum* were treated with ethanol, the blue fluorescence of the secretory cavity in the inner layer of the cork and the secretory duct of the phloem disappeared, but the blue fluorescence of the cork layer and wood was still present (Figure 2(A1,A2,C1,C2)). This indicated that the fluorescence in the secretory cavity of the inner layer of the plug and the secretory duct of the phloem was produced by coumarin. Hence, in *P. decursivum*, coumarin is mainly distributed in the secretory tissues, such as the secretory cavity and secretory duct (Figure 2(B1,B2)).

### 2.3. Selection of the Matrix

**Figure 3:**
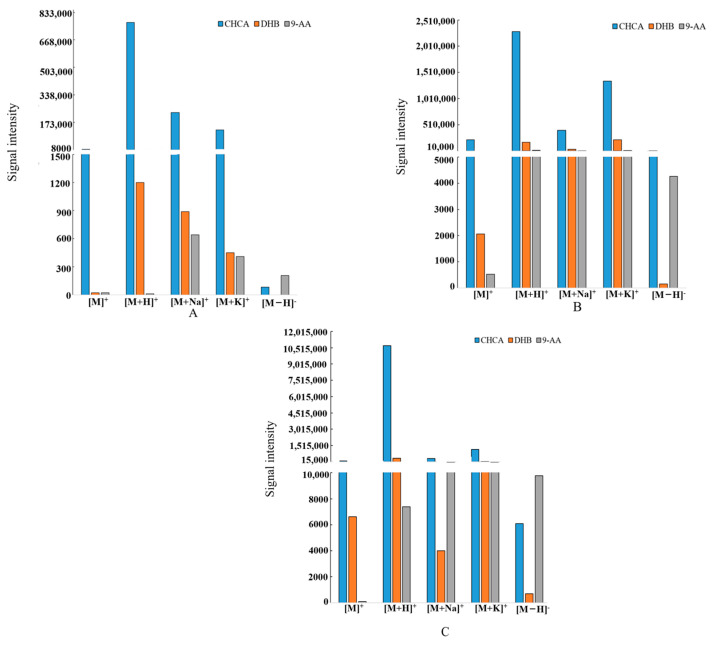
Signal intensity of representative coumarins in different matrices. (**A**) Nodakenin, (**B**) imperatorin, (**C**) oxypeucedanin.

In MALDI-TOF-MSI, there are significant differences in the resolution efficiency of different matrices, and the properties of the matrices themselves also affect the effectiveness of sample detection. Therefore, the choice of the matrix plays an important role in the analysis of compounds in a sample [31]. In the study, nodakenin, imperatorin, and oxypeucedanin were selected as representative coumarins for MALDI-TOF-MSI matrix selection; nodakenin is an indicator of the quality of *P. decursivum* [3]. Three commonly used matrices (DHB, CHCA, and 9-AA) were compared in both positive and negative ion detection modes [32,33]. The results showed that the [M]^+^, [M+H]^+^, [M+Na]^+^, and [M+K]^+^ signals of the three standards were detected in positive mode with CHCA as the matrix with high intensity (Figure 3 and Table 1). The structures of the identified coumarins are provided in the Supporting Materials (Figure S1).

### 2.4. Distribution Characteristics of Coumarins in the Root of P. decursivum

**Figure 4:**
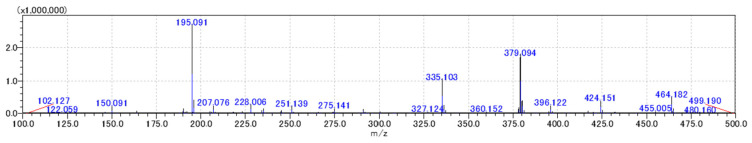
Mass spectrum of *P. decursivum* using the CHCA substrate.

Based on the above experiments, frozen sections of *P. decursivum* were analyzed by MALDI-TOF-MSI. According to the detected MS peak (Figure 4), the standard mass-to-charge ratios of coumarins in *P. decursivum* roots reported in the literature were used for comparison, and the signal peaks were assigned (Table 2). MALDI-TOF-MSI was used to detect 27 coumarins in the root of *P. decursivum*.

**Figure 5:**
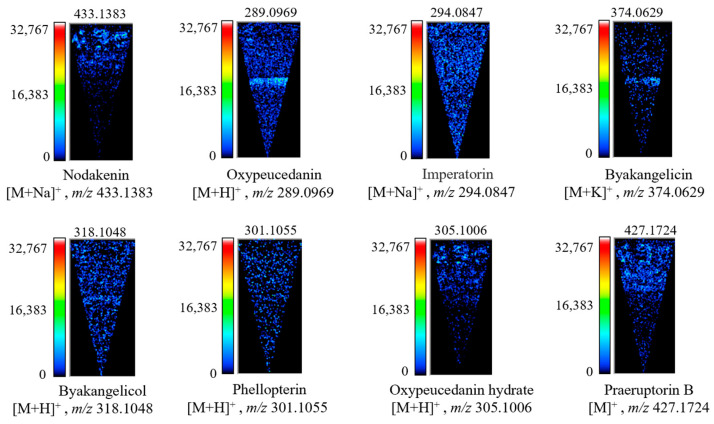
MALDI-TOF-MSI of partially representative coumarins in the root of *P. decursivum*.

In cross sections of the root of *P. decursivum*, the pith, xylem, phloem, cortex, and periderm are visible from the inside to the outside [55]. As shown in Figure 5, coumarins in *P. decursivum* were mainly distributed in the periderm, cortex, and phloem, which is consistent with the results of previous studies [56,57,58]. Imperatorin ([M + Na]^+^, *m*/*z* 294.0847) and phellopterin ([M + H]^+^, *m*/*z* 301.1055) were evenly distributed in the pith of the stem, xylem, phloem, periderm, and cortex. Oxypeucedanin ([M + H]^+^, *m*/*z* 289.0969), byakangelicin ([M + K]^+^, *m*/*z* 374.0629), and byakangelicol ([M + H]^+^, *m*/*z* 318.1048) were mainly distributed in the phloem, with less distribution in other parts. Nodakenin ([M + Na]^+^, *m*/*z* 433.1383), oxypeucedanin hydrate ([M + H]^+^, *m*/*z* 305.1006), and praeruptorin B ([M]^+^, *m*/*z* 427.1724) were mainly distributed in the cortex and periderm. The distribution of other coumarins is provided in the Supporting Materials (Figure S2).

Coumarin is a secondary metabolite derived from the phenylpropanoid pathway. Since the biosynthesis pathway of plant secondary metabolites is affected by the environment, growth period, and genes, the accumulation and distribution of secondary metabolites are affected by these factors as well [59,60]. Therefore, the distribution of coumarins in different tissues is different.

### 2.5. Network Pharmacology Research

#### 2.5.1. Active Ingredient and Disease Target Prediction

**Figure 6:**
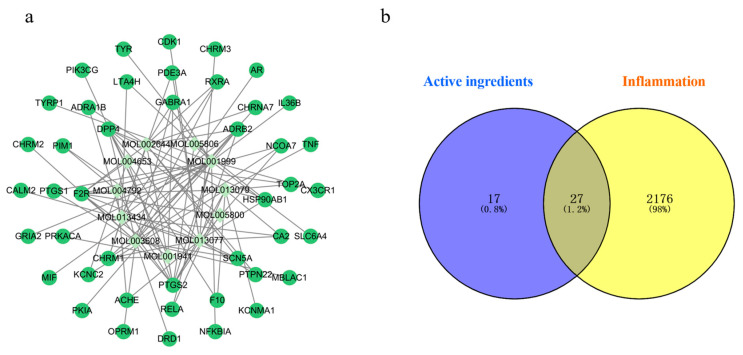
(**a**) Active ingredient targets’ interaction network, (**b**) Venn diagram.

After searching the TCMSP and Swiss Target Prediction databases, 11 ingredients were selected (Table 3) for target prediction, and a total of 44 active ingredient targets were obtained (Figure 6a). The disease targets were searched with “inflammation” as the keyword, and the targets obtained from the databases were merged and deduplicated, obtaining a total of 2203 targets. The active ingredient targets and disease targets were analyzed and represented in a Venn diagram in Venny2.1.0; a total of 27 intersection targets were obtained (Figure 6b), which are potential targets for the anti-inflammatory effect of *P. decursivum*.

#### 2.5.2. Protein–Protein Interaction (PPI) Network Analysis

**Figure 7:**
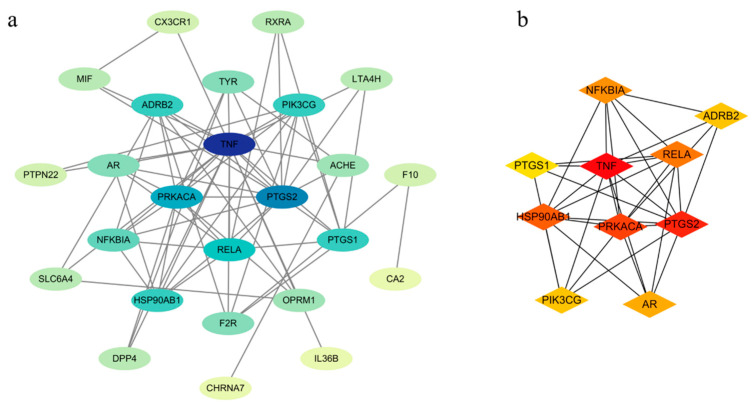
(**a**) PPI network diagram of interacting targets, (**b**) hub targets.

The 27 intersection targets obtained as described in “Section 2.5.1” were imported into the STRING database to analyze protein interactions. After the data were exported, Cytoscape was used to obtain a PPI network diagram, where the higher the interaction score, the darker the color, and the closer the interaction relationship between the examined proteins. As can be seen from Figure 7a, TNF has the darkest color, indicating that TNF is the key target responsible for the plant anti-inflammatory effects. After screening the hub targets using MCC, the top 10 targets were obtained, and the PPI network of the core targets was established (Figure 7b).

#### 2.5.3. Gene Ontology (GO) Enrichment Analysis and Kyoto Encyclopedia of Genes and Genomes (KEGG) Pathway Analysis

**Figure 8:**
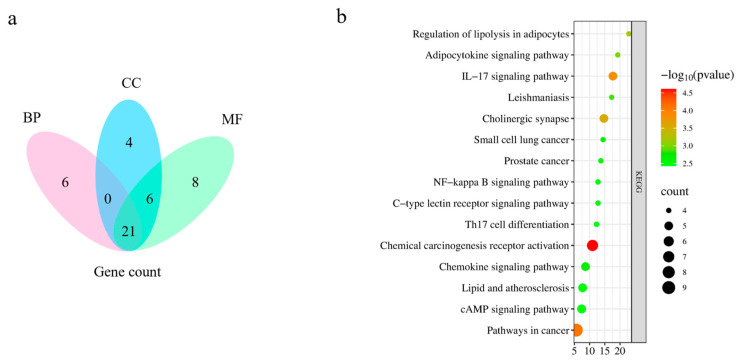
(**a**) The number of intersecting genes for BPs, CCs, and MFs in GO enrichment analysis, (**b**) KEGG enrichment analysis.

By performing a GO enrichment analysis of the intersecting targets related to *P. decursivum* active ingredient targets and anti-inflammatory disease targets, a total of 162 GO entries were obtained (*p* < 0.01). A total of 117 entries were related to biological processes (BPs), mainly involved in inflammatory response, positive regulation of protein kinase B signaling, and positive regulation of peptidyl serine physiology. Twenty-two entries were related to cellular components (CCs), mainly, the plasma membrane, neuron projections, and the cell surface. Twenty-three entries were related to molecular functions (MFs), mainly, protein homodimerization activity, identical protein binding and acetylcholine binding. According to the *p*-value, the top 10 GO enrichment analysis results are shown in Figure S3 (Supporting Materials). Based on the number of enriched genes after deleting duplicates, BPs, CCs, and MFs showed a total of 21 intersecting genes (Figure 8a). A total of 59 related pathways were identified through KEGG pathway enrichment analysis, including chemical cancer receptor activation, pathways in cancer, IL-17 signaling pathway. The top 15 pathways were selected from small to large according to the *p*-value, and a bar chart was drawn (Figure 8b).

#### 2.5.4. Molecular Docking

**Figure 9:**
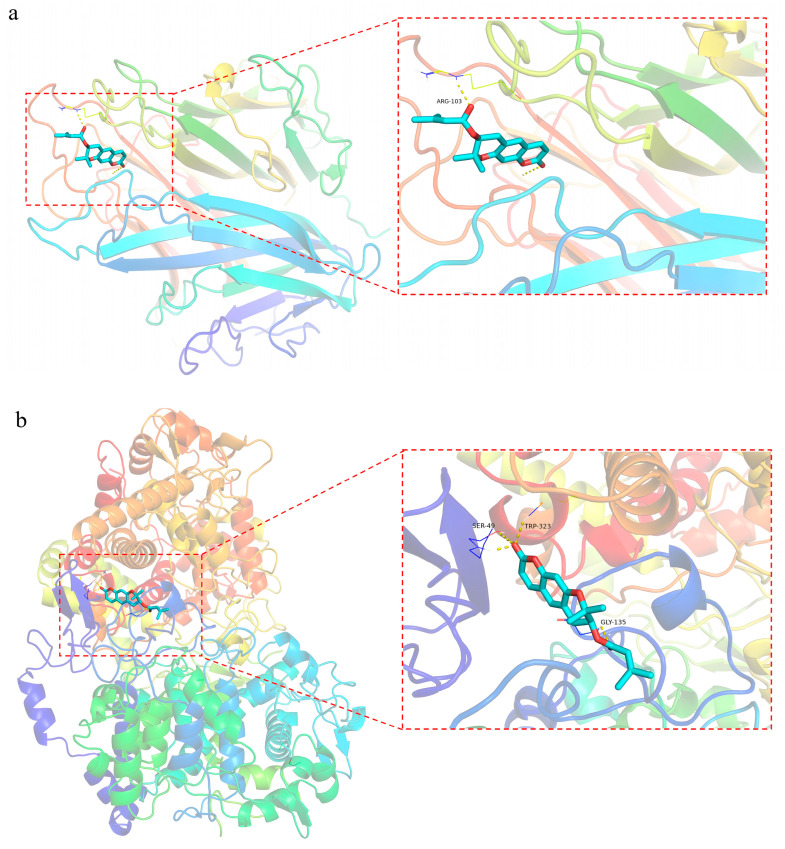

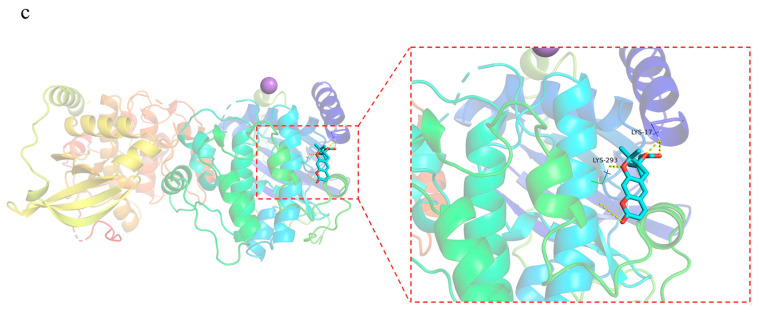
Binding conformations. (**a**) TNF–decursin; (**b**) PTGS2–decursin; (**c**) PRKACA–decursin.

Th top three active ingredients and core targets with respect to the degree of molecular docking were selected for molecular docking after MCC analysis. In general, if the binding energy between the ligand and the target protein is less than −5 kcal/mol, the binding between the ligand and the receptor protein is stable, and the lower the binding energy, the more stable the binding [61]. The molecular docking results showed binding energies of less than −5 kcal/mol, as shown in Table 4. Decursin had the strongest binding affinity with TNF, PTGS2, and PRKACA, leading to the strongest binding stability. The docking results are shown in Figure 9.

## 3. Materials and Methods

### 3.1. Materials and Reagents

The plant materials of *P. decursivum* used in this experiment were fresh and were acquired from Dashui Village, Luoba Town, Shixing County, Shaoguan City, Guangdong Province (24°43′25.73″ N, 114°15′16.14″ E) in May. They were identified by Professor Chen Yuan from the Department of Traditional Chinese Medicine Cultivation and Identification at Gansu Agricultural University and confirmed as a plant material of *P. decursivum*. A voucher specimen (No. GAUAB-PD-20230518) was deposited in the herbarium of the Department of Chinese herbal medicine, Agronomy building of Gansu Agricultural University, Lanzhou, China.

An electric-heating constant-temperature vacuum-drying oven (Shanghai Yuejin Medical Equipment Co., Ltd., Shanghai, China, model: DZF-1B), a freezing microtome (Leica Company, Wetzlar, Germany, model: CM1950), an upright fluorescence microscope (Gansu Jiarui Trading Co., Ltd., Baiyin, China), an upright inverted integrated fluorescence microscope (ECHO Company, Zurich, IL, USA, model: BX61+DP70, RVL100-G), glycerol (China National Pharmaceutical Group Chemical Reagent Co., Ltd., Shanghai, China, batch number: 20201116), OCT embedding agent (Leica Company, Germany, batch number: 14020108926), nodakenin (20070604, ≥98%), imperatorin (18051502, ≥98%), and oxypeucedanin (y27s9s65152, purity ≥ 98.0%) were used. An imaging mass spectrometer (Shimadzu iMScope TRIO, Kyoto, Japan), the fully automatic matrix spray iMLayer (Shimadzu, Kyoto, Japan), conductive glass slides (Bruker, Bremen, Germany), and a vacuum dryer (Shanghai Yueci Electronic Technology Co., Ltd., Shanghai, China) were also employed.

### 3.2. Fluorescence Imaging Technology for Tissue Distribution of Coumarins in the Root of P. decursivum

#### 3.2.1. Selection of Frozen Section Thickness

The slicing method was described in our previous work [62]. The fresh root of *P. decursivum* was washed and cut into small sections of about 0.5 cm, which were placed in 15% glycerol and vacuumed until they sank to the bottom of the bottle. The glycerol solution on the surface was then removed, and the small segments of *P. decursivum* were precooled in a freezing microtome for 30 min, immersed in OCT embedding medium on a precooled tray, frozen until the OCT medium turned white, and then sliced on a freezing microtome (CM1950, Leica, Germany). The sections were then quickly frozen at −22 °C for 10 min, further cut into tissue sections with thicknesses of 20 µm, 25 µm, 30 µm, 35 µm, and 40 µm, attached to glass slides, sealed with the 15% glycerol solution, and observed under an upright microscope to see if there were bubbles in the tissue. If there were bubbles, a dropper was used to gently expel the bubbles. After the bubbles were completely expelled, a coverslip was placed on the tissue, and its integrity and stretching level were observed in bright field using an upright and inverted fluorescence microscope; pictures were taken.

#### 3.2.2. Localization and Observation of Coumarins

Coumarins usually produce blue fluorescence under ultraviolet light and are easily soluble in methanol, toluene, ether, and other organic solvents [58,63]. Hence, the sections were fixed with 95% ethanol for 30 min to remove coumarins and then sealed and observed using the DAPI channel of an upright and inverted fluorescence microscope.

### 3.3. Study on the Spatial Distribution of Coumarins in the Root of P. decursivum Using MALDI-TOF-MSI

#### 3.3.1. Preparation of Standard Solutions and Screening of Matrices

The reference standards of nodakenin, imperatorin, and oxypeucedanin were accurately weighed and dissolved in a 70% methanol aqueous solution and then ultrasonically treated for 5 min. Finally, 1 mg/mL standard solutions were prepared. The prepared standard solutions (1 μL) were dripped onto the target plates. After the samples had dried, the matrix solution (1 μL) was dripped to cover the samples. MALDI-TOF-MSI analysis was carried out after drying.

CHCA (α-cyano-4-hydroxycinnamic acid), DHB (2,5-dihydroxybenzoic acid), and 9-AA (9-aminoacridine) were accurately weighed and dissolved in a 70% methanol aqueous solution; then, 10 mg/mL matrix solutions were prepared.

#### 3.3.2. Spraying of the Substrate

The substrate was sublimated using the substrate sublimation device iMLayer (Shimadzu, Kyoto, Japan) and sprayed under the following conditions: the thickness of DHB (300 mg) at 180 °C was 0.5 μm, the thickness of 9-AA (300 mg) at 220 °C was 0.5 μm, and the thickness of CHCA (300 mg) at 250 °C was 0.5 μm. The tissue was sprayed and sliced.

#### 3.3.3. Sample Preparation

The slicing method is described in “Section 3.2.1”. The selected slice thickness was 30 μm. The slices were placed on conductive slides (made of indium tin oxide) in a vacuum dryer for 20 min.

#### 3.3.4. MALDI-TOF-MSI

**Figure 10:**
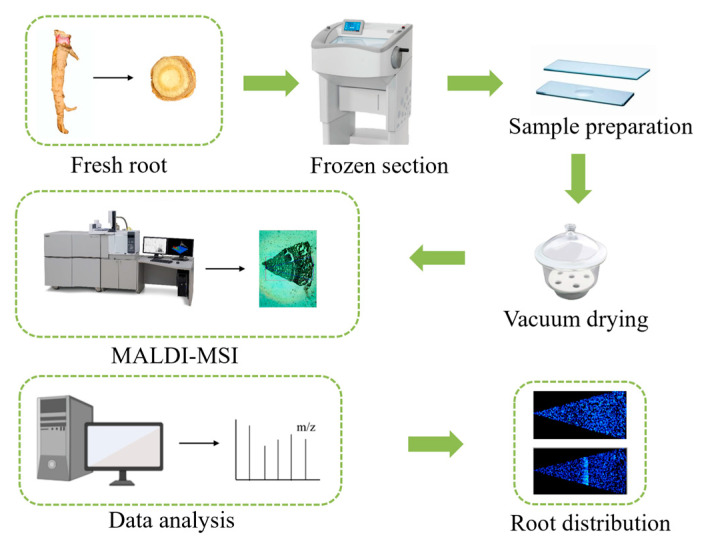
Schematic diagram of the MALDI-TOF-MSI program for imaging coumarins of *P. decursivum*.

MALDI-TOF-MSI data were acquired by using an imaging mass spectrometer (iMScope TRIO, Shimadzu) equipped with an internal optional microscope and a laser source (Nd: YAG at 355 nm). Molecular images were recorded and analyzed by imaging MS Solution 1.30 (Shimadzu). Ions of *m*/*z* 100–500 were measured in positive and negative ionization modes. The laser diameter was set to 1 (~10 μms, an arbitrary unit of iMScope). Other parameters of MALDI-TOF-MSI were the following: detector voltage 1.87 kV, sample voltage 3.50 kV, accumulation 1 time/pixel, laser repetition rate 1000 Hz, laser shots 80, pitch 35 × 35 μm^2^, and laser intensity 25 (arbitrary unit of iMScope). The MALDI-TOF-MSI operation flowchart was shown in Figure 10.

### 3.4. Network Pharmacology

#### 3.4.1. Screening of Active Ingredients

Based on the results of the MALDI-TOF-MSI studies, the top 13 components in terms of signal intensity were selected as the main active ingredients for the network pharmacology studies (Table 5).

#### 3.4.2. Target Prediction

The TCMSP (https://tcmsp-e.com/, accessed on 20 June 2023), Swiss Target Prediction (http://www.swisstargetprediction.ch, accessed on 20 June 2023), GeneCards (https://www.genecards.org/, accessed on 20 June 2023), and OMIM (https://omim.org/, accessed on 20 June 2023) databases were used to predict the active ingredient targets and disease targets.

#### 3.4.3. Construction of a PPI Network Diagram and Screening of the Core Targets

The online tool VENNY2.1 (https://bioinfogp.cnb.csic.es/tools/venny/, accessed on 21 June 2023) was used to intersect the active ingredient targets and anti-inflammatory disease targets and obtain potential anti-inflammatory targets. Then, they were imported into the STRING database (https://www.stringdb.org/, accessed on 21 June 2023), searching the species “Homo sapiens”, with the minimum interaction score of “≥0.400”. MCC was used to screen out the top 10 core targets based on the relationship between nodes and edges. 

#### 3.4.4. GO Enrichment Analysis and KEGG Pathway Analysis

GO enrichment analysis is used to explore potential biomolecular mechanisms, which include BP, CC, and MF. KEGG pathway analysis was also used to identify biological functions and candidate targets. David database (https://david.ncifcrf.gov/home.jsp, accessed on 22 June 2023) was used to GO enrichment analysis and KEGG pathway analysis on intersecting targets.

#### 3.4.5. Molecular Docking

The molecular docking technology combines active ingredients with target proteins to verify the prediction results of network pharmacology in a virtual evaluation manner [64]. The target protein crystal structures corresponding to the screened key targets were found in the protein structure database RCSB (https://www.rcsb.org/, accessed on 24 June 2023) in pdb format, and the protein crystals were processed using PyMol 2.3.4 software to separate their respective original ligands. The active ingredient, target protein crystal structure, and original ligand were processed using Autodock Tools 1.5.6 software and saved in pdbqt format. Then, the “grid box” of the target protein and the original ligand was found as the active pocket, and verification of the original ligand docking was performed. Autodock Vina 1.5.6 was used to dock the target protein and the active ingredient, and the appropriate conformation was selected. PyMol 2.3.4 software was used for visualization based on the docking results.

## 4. Conclusions

Coumarin is one of the important secondary metabolites of *P. decursivum*. Previous studies have shown that coumarin has anti-inflammatory, anti-tumor, and other pharmacological effects. The secondary metabolites of medicinal plants are the basis for their medicinal effects; so, metabolites play a vital role in traditional Chinese medicine. In this study, MALDI-TOF-MSI was used for the first time to elucidate the spatial distribution of coumarins in the root tissue of *P. decursivum*, and combined network pharmacology and molecular docking were employed to predict the potential targets and pathways at the basis of *P. decursivum* anti-inflammatory effects. Our research showed that frozen sections of 30 µm thickness were conducive to observing the distribution of coumarins in the root of *P. decursivum*. After the optimization of matrix and mode, we chose to analyze the samples in the positive mode, with CHCA as the matrix. Through the attribution of signal peaks, a total of 27 coumarins were identified in the roots of *P. decursivum*, which appeared to be mainly stored in the phloem, cortex, and periderm. This is consistent with previous research that indicated that coumarins were mainly concentrated in the cortex. Based on the results of MALDI-TOF-MSI, the components with the highest signal intensity (including nodakenin, imperatorin, decursin, etc.) were screened in network pharmacology and molecular docking studies. The results showed that a total of 27 targets contributed to the plant’s effects. Key targets such as TNF, PTGS2, PRKACA, HSP90AB1, RELA, and NFKBIA appeared to be involved in chemical carcinogenesis–receptor activation, pathways in cancer, IL-17 signaling pathway, cholinergic synapse pathway, and regulation of lipolysis in adipocytes. TNF is mainly secreted by macrophages, can induce cell death in certain tumor cell lines, and is mainly involved in the inflammatory response. It can enhance infection resistance by activating neutrophils and platelets, enhancing the killing ability of macrophages/NK cells, and stimulating the immune system. It can also play a pathological role in various autoimmune diseases and processes, such as graft-versus-host rejection and rheumatoid arthritis [65]. PTGS2 is a key molecule with anti-inflammatory and analgesic effects, playing a crucial role in biological processes such as inflammatory response, inflammation-related gene expression, cell apoptosis, and immune response [66]. PRKACA is a downstream molecule of the second messenger cAMP. The activated cAMP/PRKACA pathway can inhibit the inflammatory response [67]. Molecular docking showed that the binding energies of the core active ingredients and the core target proteins (TNF, PTGS2, and PRKACA) were all < −5.0 kcal/mol, which proved that they had stable binding activities, and decursin exhibited the most stable interaction.

This study integrated multiple disciplines such as histochemistry, computer informatics, and pharmacology to establish a method for the visual characterization of the tissue localization of coumarins and the analysis of their pharmacological action mechanism, which can provide a theoretical basis for the quality evaluation and clinical use of *P. decursivum*. It can also provide a reference for research on metabolites of other medicinal plants.

## Figures and Tables

**Table 1:** Signal intensity of representative coumarins in different matrices of *P. decursivum*.

Standard Compound	Matrix	Ionic Mode	Relative Ionic Intensities (%)
Nodakenin	CHCA	positive	11,199
		negative	32
	DHB	positive	22
		negative	--
	9-AA	positive	21
		negative	27
Imperatorin	CHCA	positive	223,641
		negative	949
	DHB	positive	2064
		negative	49
	9-AA	positive	521
		negative	1620
Oxypeucedanin	CHCA	positive	101,031
		negative	3488
	DHB	positive	6623
		negative	174
	9-AA	positive	83
		negative	36

**Table 2:** Tentatively identified coumarins from the roots *P. decursivum* with MALDI-MSI.

Compound (or Its Isomer)	Molecular Formula	Adduct Ions	Theoretical (*m*/*z*)	Observed (*m*/*z*)	Error (ppm)	Ref.
Nodakenin	C_20_H_24_O_9_	[M + Na]^+^	433.1385	433.1383	−0.46	[34]
Oxypeucedanin	C_16_H_14_O_5_	[M + H]^+^	289.0987	289.0969	−6.23	[35]
Imperatorin	C_16_H_14_O_4_	[M + Na]^+^	294.0824	294.0847	7.82	[35]
Byakangelicol	C_17_H_16_O_6_	[M + H]^+^	318.1059	318.1048	−3.46	[35]
Phellopterin	C_17_H_16_O_5_	[M + H]^+^	301.1077	301.1055	−7.31	[36]
Oxypeucedanin hydrate	C_16_H_16_O_6_	[M + H]^+^	305.1026	305.1066	−6.56	[37]
Isopimpinellin	C_13_H_10_O_5_	[M + K]^+^	286.0199	286.0150	−17.13	[38]
Praeruptorin B	C_24_H_26_O_7_	[M]^+^	427.1712	427.1724	2.81	[39]
Praeruptorin A	C_21_H_22_O_7_	[M + H]^+^	387.1445	387.1453	2.07	[40]
Osthenol	C_14_H_14_O_3_	[M]^+^	232.1010	232.0963	−20.25	[41]
Scopolin	C_16_H_18_O_9_	[M + Na]^+^	377.0849	377.0789	−15.91	[42]
Isomeranzin	C_15_H_16_O_4_	[M + Na]^+^	283.0947	232.0963	7.78	[43]
Byakangelicin	C_17_H_18_O_7_	[M + K]^+^	374.0723	374.0629	−2.51	[44]
Dehydrogeijerin	C_15_H_14_O_4_	[M + H]^+^	259.0971	259.1008	14.28	[45]
Decursin	C_19_H_20_O_5_	[M + H]^+^	329.1344	329.1328	−4.86	[46]
Scoparone	C_11_H_10_O_4_	[M + H]^+^	208.0692	208.0702	4.81	[47]
Herniarin	C_10_H_8_O_3_	[M + H]^+^	177.0552	177.0583	17.51	[38]
Columbianetin acetate	C_16_H_16_O_5_	[M]^+^	290.1040	290.1017	−7.93	[48]
Umbelliprenine	C_24_H_30_O_3_	[M + K]^+^	405.1832	405.1748	−20.73	[49]
Oxypeucedanin hydrate-3″-ethyl ether	C_18_H_20_O_6_	[M + Na]^+^	355.1158	355.1184	7.32	[50]
Angelol A	C_20_H_24_O_7_	[M + Na]^+^	400.1454	400.1477	5.75	[51]
Pabulenol	C_17_H_16_O_4_	[M + H]^+^	286.1161	286.1147	−4.89	[51]
Apiosylskimmin	C_20_H_24_O_12_	[M + H]^+^	459.1414	459.1455	8.93	[52]
Auraptene	C_19_H_22_O_3_	[M + K]^+^	338.1239	338.1214	−7.39	[53]
Scopoletin	C_10_H_8_O_4_	[M + K]^+^	231.0060	231.0059	−0.43	[54]
Angenomalin	C_14_H_12_O_3_	[M + H]^+^	230.0899	230.0894	−2.17	[51]
(+)-Decursinol	C_14_H_14_O_4_	[M]^+^	246.0892	246.0882	−4.06	[45]

**Table 3:** Active ingredient information for target prediction.

MOL ID	MOL Name	CAS
MOL001999	Scoparone	120-08-1
MOL013434	Auraptene	495-02-3
MOL004653	Praeruptorin B	81740-07-0
MOL013079	Praeruptorin A	73069-25-7
MOL005800	Byakangelicol	26091-79-2
MOL005806	Oxypeucedanin hydrate	133164-11-1
MOL003608	Columbianetin acetate	23180-65-6
MOL013077	Decursin	5928-25-6
MOL004792	Nodakenin	495-31-8
MOL001941	Imperatorin	482-44-0
MOL002644	Phellopterin	2543-94-4

**Table 4:** Binding energy.

Targets		Binding Energy (kcal/mol)	
	Scoparone	Decursin	Columbianetin Acetate
TNF	−6.4	−7.8	−5.8
PTGS2	−7.3	−8.6	−7.5
PRKACA	−5.5	−7.9	−6.8

**Table 5:** Signal intensity of the active ingredients.

No.	Ingredients	Molecular Weight	Signal Intensity
**1**	Scopaone	208.0702	31,187
**2**	Oxypeucedanin	289.0969	18,697
**3**	Auraptene	338.1214	10,089
**4**	Praeruptorin B	427.1724	9969
**5**	Praeruptorin A	387.1453	8560
**6**	(±)-Decursinol	246.0882	8334
**7**	Byakangelicol	318.1048	7330
**8**	Oxypeucedanin hydrate	305.1006	6887
**9**	Columbianetin acetate	290.1017	6299
**10**	Decursin	329.1328	6033
**11**	Nodakenin	433.1383	5930
**12**	Imperatorin	294.0847	3593
**13**	Phellopterin	301.1055	3269

## Data Availability

The data presented in this study are available on request from the corresponding author.

## Supplementary Materials

The following supporting information can be downloaded at: https://www.mdpi.com/article/10.3390/molecules29143346/s1, Figure S1. The structural formula of the compound. Figure S2. MALDI-TOF-MSI of coumarins in the root of *P. decursivum*. Figure S3. GO enrichment analysis results.
