# Supplementary material for: Study on the Anti-Inflammatory Mechanism of Coumarins in Peucedanum decursivum Based on Spatial Metabolomics Combined with Network Pharmacology
Source: Molecules. 2024 Jul 17;29(14):3346. doi: 10.3390/molecules29143346 (PMC11280318; doi:10.3390/molecules29143346)
Supplement: Supplementary file 1 [file molecules-29-03346-s001.zip › molecules-3063067-supplementary.pdf]

Supplementary Materials

Study on the Anti-Inflammatory Mechanism of Coumarins in *Peucedanum decursivum* Based on Spatial Metabolomics Combined with Network Pharmacology

Zeyu Li and Qian Li *
State Key Laboratory of Aridland Crop Science, College of Agronomy, Gansu Agricultural University, Lanzhou 730070, China; lizy092023@163.com
* Correspondence: liqian1984@gsau.edu.cn

1. The structural formula identified by MAIDL-TOF-MSI are as follows:

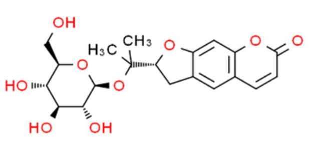
Nodakenin

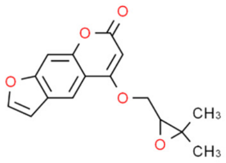
Oxypeucedanin

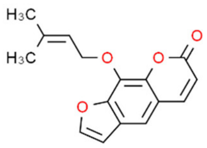
Imperatorin

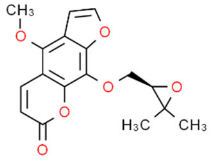
Byakangelicol

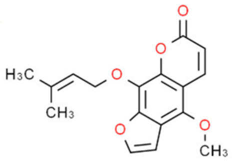
Phellopterin

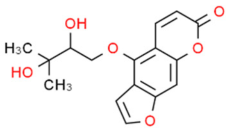
Oxypeucedanin hydrate

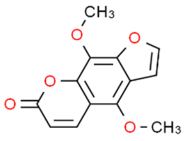
Isopimpinellin

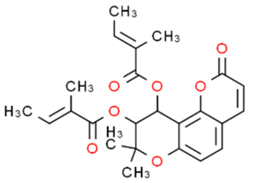
Praeruptorin B

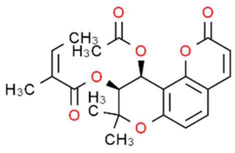
Praeruptorin A

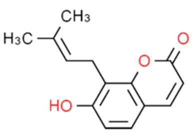
Osthenol

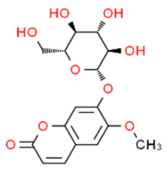
Scopolin

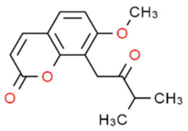
Isomeranzin

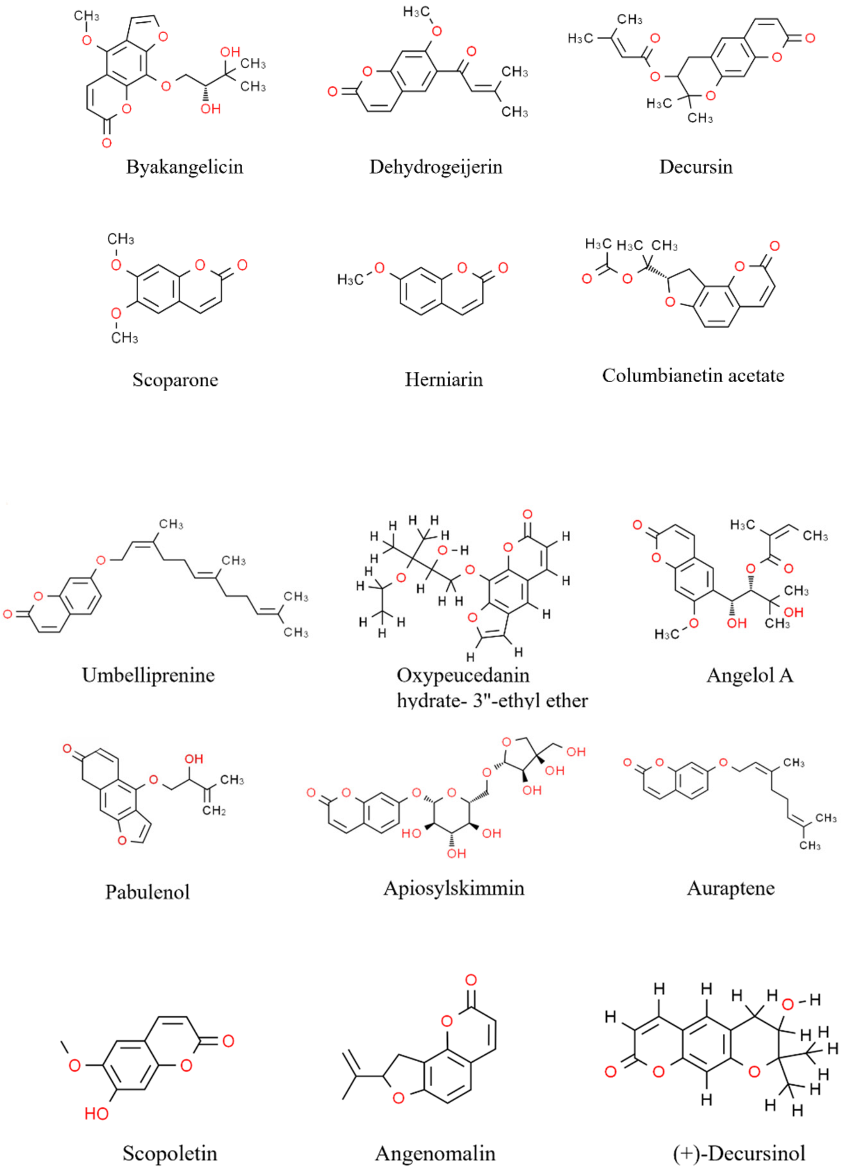

Figure S1. The structural formula of the compound.

2. The distribution of MALDI-TOF-MSI compounds is as follows:

Angelol A ([M+Na]$^+$, *m/z* 400.1477), decursin ([M+H]$^+$, *m/z* 329.1328), and scoparone ([M+H]$^+$, *m/z* 208.0702) were mainly distributed in the phloem, cortex, and periderm. Scopolin([M+Na]$^+$, *m/z* 377.0789), oxypeucedanin hydrate- 3''-ethyl ether([M+Na]$^+$, *m/z* 355.1184), auraptene([M+K]$^+$, *m/z* 338.1214), dehydrogeijerin([M+H]$^+$, *m/z* 259.1008), and herniarin([M+H]$^+$, *m/z* 177.0583) were mainly distributed in the phloem and cortex. Umbelliprenine([M+K]$^+$, *m/z* 405.1748), praeruptorin A([M+H]$^+$, *m/z* 387.1453) and (+)-decursinol([M]$^+$, *m/z* 246.0882) were mainly distributed in the cortex and periderm. Pabulenol([M+H]$^+$, *m/z* 286.1147) was mainly distributed in the cortex. Apiosylskimmin([M+H]$^+$, *m/z* 459.1455), columbianetin acetate([M]$^+$, *m/z* 290.1017), isopimpinellin([M+K]$^+$, *m/z* 286.0150), isomeranzin([M+Na]$^+$, *m/z* 400.1477), osthenol([M]$^+$, *m/z* 232.0963), scopoletin([M+K]$^+$, *m/z* 231.0059), and angenomalin([M+H]$^+$, *m/z* 230.0894)

were mainly distributed in the phloem.

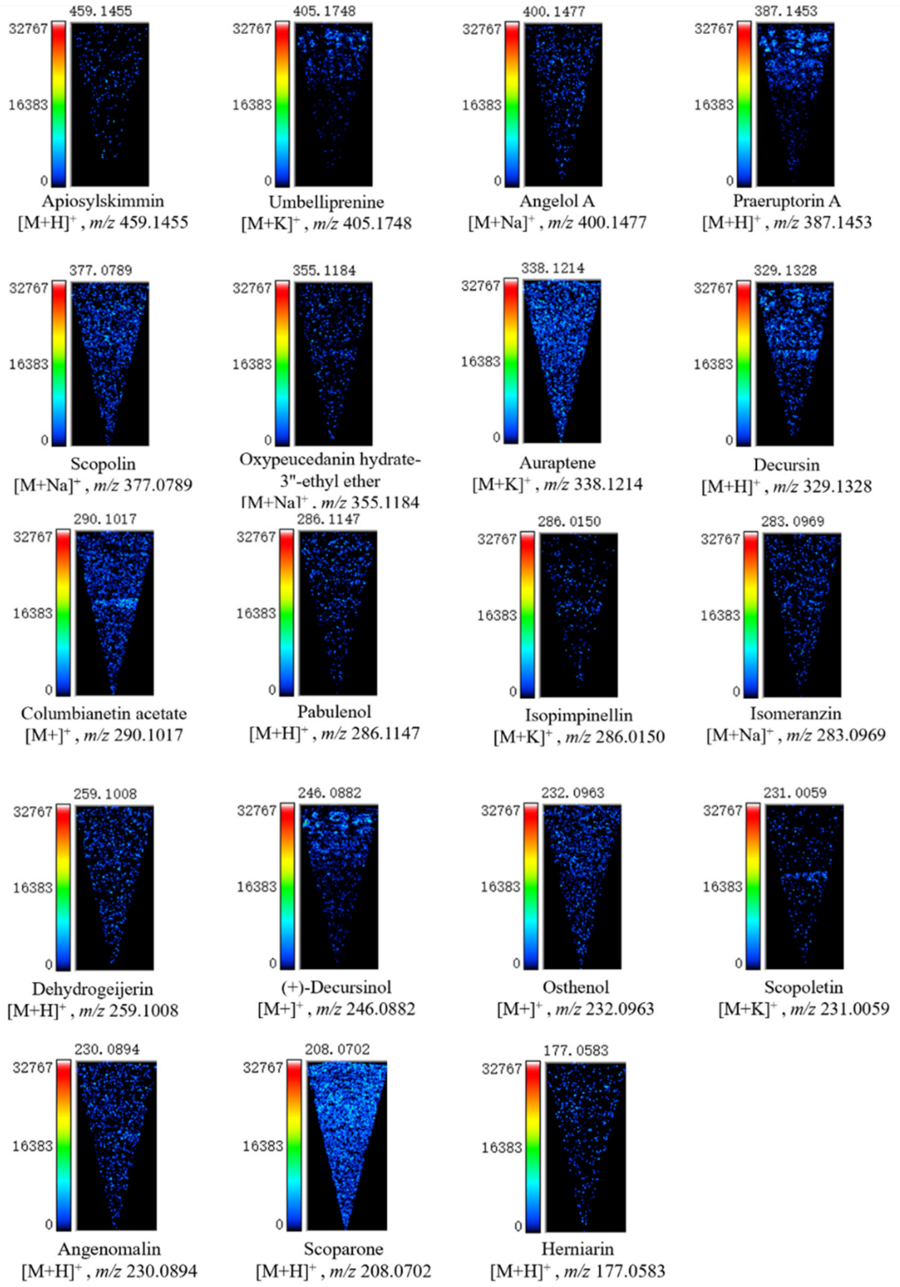

Figure S2. MALDI-TOF-MSI of coumarins in the root of *P. decursivum*.

3. According to *p*-value, the top 10 GO enrichment analysis results.

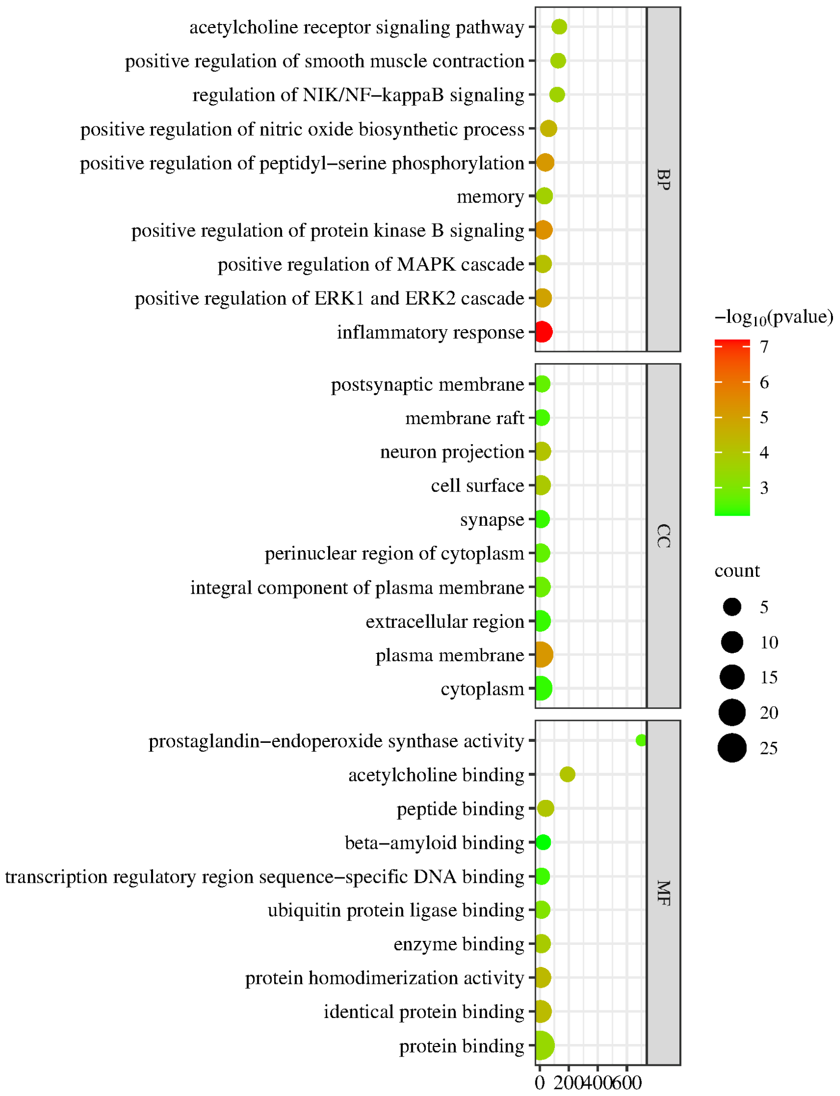

Figure S3. GO enrichment analysis results.
